# 2-Bromo­maleic acid

**DOI:** 10.1107/S1600536809029602

**Published:** 2009-08-26

**Authors:** Andreas Fischer

**Affiliations:** a Inorganic Chemistry, School of Chemical Science and Engineering, Royal Institute of Technology (KTH), 100 44 Stockholm, Sweden

## Abstract

The title compound, C_4_H_3_BrO_4_, was obtained from a solution of *meso*-2,3-dibromo­succinic acid and vanadium(IV) oxide. The crystals are isostructural with chloro­maleic acid and the mol­ecule has two geometrically different carboxyl groups, one of which has delocalized C—O bonds and is essentially coplanar with the olefinic bond plane [give dihedral angle 15.08 (16)°], whereas the other has a localized C=O bond and forms a dihedral angle of 99.6 (3)° with the C=C bond plane. Two symmetry-independent O—H⋯O hydrogen bonds link the mol­ecules into layers parallel to the *bc* plane.

## Related literature

For the structure of chloro­maleic acid, see: Wong *et al.* (2006 ▶). For the synthesis and structure of 2-bromo­fumaric acid, see: Fischer (2006 ▶). For the structure and polymorphism of maleic acid, see: Day *et al.* (2006 ▶). For the structure of 2-methyl­maleic acid, see: Batchelor & Jones (1998 ▶).
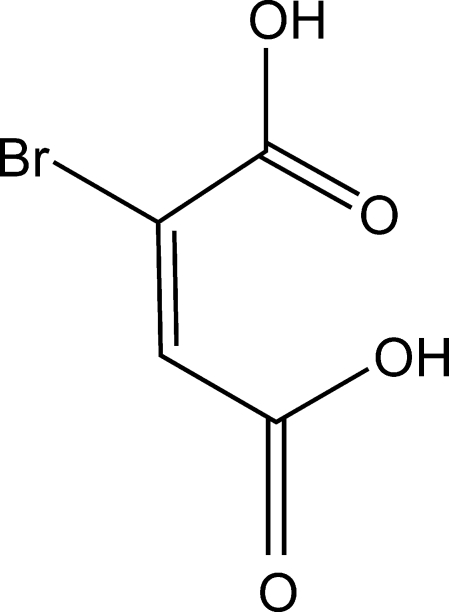


## Experimental

### 

#### Crystal data

C_4_H_3_BrO_4_
*M* *_r_* = 194.97
Monoclinic, 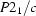

*a* = 7.5074 (12) Å
*b* = 4.9272 (6) Å
*c* = 16.966 (4) Å
β = 94.213 (12)°
*V* = 625.9 (2) Å^3^
*Z* = 4
Mo *K*α radiation
μ = 6.50 mm^−1^
*T* = 299 K
0.15 × 0.13 × 0.10 mm

#### Data collection

Bruker–Nonius KappaCCD diffractometer
Absorption correction: numerical *HABITUS* (Herrendorf & Bärnighausen, 1997 ▶) *T*_min_ = 0.325, *T*_max_ = 0.455
8638 measured reflections
1420 independent reflections
1046 reflections with *I* > 2σ(*I*)
*R*_int_ = 0.064

#### Refinement

*R*[*F* ^2^ > 2σ(*F* ^2^)] = 0.042
*wR*(*F* ^2^) = 0.075
*S* = 1.16
1420 reflections
84 parameters
H-atom parameters constrained
Δρ_max_ = 0.63 e Å^−3^
Δρ_min_ = −0.43 e Å^−3^

### 

Data collection: *COLLECT* (Nonius, 1999 ▶); cell refinement: *DIRAX* (Duisenberg, 1992 ▶); data reduction: *EVALCCD* (Duisenberg *et al.*, 2003 ▶); program(s) used to solve structure: *SHELXS97* (Sheldrick, 2008 ▶); program(s) used to refine structure: *SHELXL97* (Sheldrick, 2008 ▶); molecular graphics: *DIAMOND* (Brandenburg, 1999 ▶); software used to prepare material for publication: *publCIF* (Westrip, 2009 ▶).

## Supplementary Material

Crystal structure: contains datablocks global, I. DOI: 10.1107/S1600536809029602/ya2098sup1.cif

Structure factors: contains datablocks I. DOI: 10.1107/S1600536809029602/ya2098Isup2.hkl

Additional supplementary materials:  crystallographic information; 3D view; checkCIF report

## Figures and Tables

**Table 1:** Hydrogen-bond geometry (Å, °)

*D*—H⋯*A*	*D*—H	H⋯*A*	*D*⋯*A*	*D*—H⋯*A*
O3—H3⋯O4^i^	0.82	1.80	2.617 (4)	171
O2—H2⋯O1^ii^	0.82	1.86	2.681 (4)	176

Symmetry codes: (i) 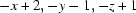; (ii) 
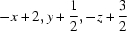
.

**Fig. 1.**
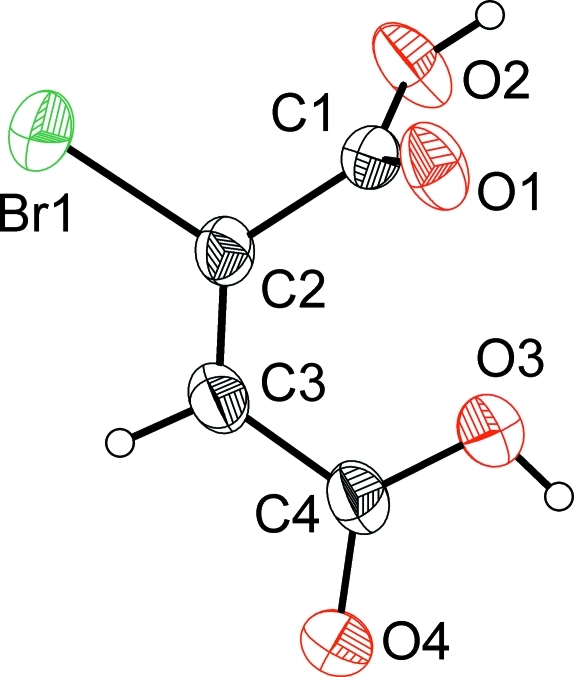
Molecular structure of the title compound. Displacement ellipsoids are drawn at the 50% probability level; H atoms are drawn as small circles of arbitrary radius.

**Fig. 2.**
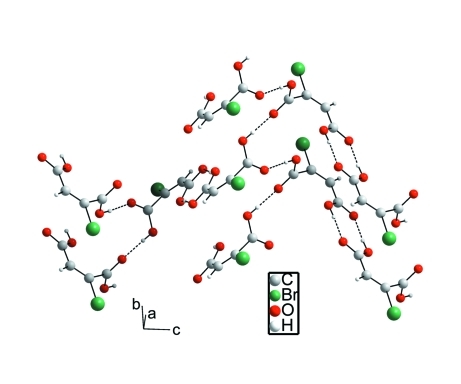
Crystal packing of the title compound viewed down the a axis. Hydrogen bonds are drawn as dashed lines.

## supplementary crystallographic information

### Comment

During the ongoing investigation of the bromo-substituted dicarboxylic acids with four carbon atoms, our group tried to prepare and characterize pure acids as well as their metal salts. One of these syntheses involved the reaction of vanadium(IV) oxide with *meso*-dibromosuccinic acid. It had been shown earlier (Fischer, 2006), that hydrogen bromide can easily be eliminated from racemic 2,3-dibromosuccinic acid, yielding 2-bromofumaric acid. Elimination of hydrogen bromide from *meso*-2,3-dibromosuccinic acid does not occur as easily. However in the presence of strong bases and at elevated temperature, elimination is observed; it yields 2-bromomaleic acid, whose structure is described here.

While most of bond lengths and angles in the molecule of 2-bromomaleic acid (Fig. 1) are close to those found in 2-bromofumaric acid (Fischer, 2006) and unsubstituted maleic acid (Day *et al.*, 2006), the title compound, in contrast to the aforementioned molecules, is essentially non-planar. In fact, two carboxylic groups in 2-bromomaleic acid form dihedral angle of 77.6 (3)° with each other and only one of them (O3–C4–O4) is almost coplanar with the C2=C3 double bond plane, whereas the second one (O1–C1–O2) forms with the latter plane dihedral angle of 99.6 (3)° The carboxylic group, which is almost coplanar with the olefinic bond, shows much higher degree of delocalization (C4–O3 1.269 (4) and C4–O4 1.254 (5) Å), than the second carboxylic group, bonded to the bromo-substituted carbon atom (C1–O1 1.197 (4) and C1–O2 1.299 (5) °). It is noteworthy that similar geometrical peculiarities were observed in other known structures of monosubstituted maleic acid derivatives, namely in 2-chloromaleic acid (Wong *et al.*, 2006), isostructural with the title compound, and 2-methylmaleic acid (Batchelor & Jones, 1998).

There are two symmetry independent O—H..O bonds (Table 1), one of which involves delocalized carboxyl group and is responsible for formation of dimeric centrosymmetric motives traditional to carboxylic acid crystal structures. Another H-bond involves non-symmetric carboxylic group and further links dimeric aggregates into layers parallel to the *bc*-plane (Fig. 2).

### Experimental

89 mg of VO_2_ (AlfaAesar, 99%), was added to a solution of 270 mg of *meso*-dibromosuccinic acid (Sigma Aldrich, 98%) in 4.2 ml of demineralized water. Upon heating to 90°C, vanadium oxide got dissolved, yielding a dark-blue solution, which was put aside for evaporation. Within a week, colourless crystals of the title compound were obtained.

### Refinement

H atoms could be located in the Fourier map, however, their isotropic refinement did not yield satisfactory *X*–H distances. Therefore, H atoms were placed at calculated positions with d(C–H)=0.93 Å, d(O–H)=0.82 Å and included in the subsequent refinement in riding motion approximation with *U*_iso_=1.2U_eq _of the carrier atom (1.5 *U*_eq_ for hydroxyl H atoms).

### Crystal data

**Table:**

C_4_H_3_BrO_4_	*F*(000) = 376
*M**_r_* = 194.97	*D*_x_ = 2.069 Mg m^−^^3^
Monoclinic, *P*2_1_/*c*	Mo *K*α radiation, λ = 0.71073 Å
Hall symbol: -P 2ybc	Cell parameters from 28 reflections
*a* = 7.5074 (12) Å	θ = 5.6–19.2°
*b* = 4.9272 (6) Å	µ = 6.50 mm^−^^1^
*c* = 16.966 (4) Å	*T* = 299 K
β = 94.213 (12)°	Block, colourless
*V* = 625.9 (2) Å^3^	0.15 × 0.13 × 0.10 mm
*Z* = 4	

### Data collection

**Table:**

Bruker–Nonius KappaCCD diffractometer	1046 reflections with *I* > 2σ(*I*)
Radiation source: fine-focus sealed tube	*R*_int_ = 0.064
φ and ω scans	θ_max_ = 27.5°, θ_min_ = 4.7°
Absorption correction: numerical *HABITUS* (Herrendorf & Bärnighausen, 1997)	*h* = −9→9
*T*_min_ = 0.325, *T*_max_ = 0.455	*k* = −6→6
8638 measured reflections	*l* = −17→22
1420 independent reflections	

### Refinement

**Table:**

Refinement on *F*^2^	Primary atom site location: structure-invariant direct methods
Least-squares matrix: full	Secondary atom site location: difference Fourier map
*R*[*F*^2^ > 2σ(*F*^2^)] = 0.042	H-atom parameters constrained
*wR*(*F*^2^) = 0.075	*w* = 1/[σ^2^(*F*_o_^2^) + (0.0113*P*)^2^ + 1.1107*P*] where *P* = (*F*_o_^2^ + 2*F*_c_^2^)/3
*S* = 1.16	(Δ/σ)_max_ = 0.001
1420 reflections	Δρ_max_ = 0.63 e Å^−^^3^
84 parameters	Δρ_min_ = −0.43 e Å^−^^3^
0 restraints	

### Special details

**Table:**

Geometry. All e.s.d.'s (except the e.s.d. in the dihedral angle between two l.s. planes) are estimated using the full covariance matrix. The cell e.s.d.'s are taken into account individually in the estimation of e.s.d.'s in distances, angles and torsion angles; correlations between e.s.d.'s in cell parameters are only used when they are defined by crystal symmetry. An approximate (isotropic) treatment of cell e.s.d.'s is used for estimating e.s.d.'s involving l.s. planes.
Refinement. Refinement of *F*^2^ against ALL reflections. The weighted *R*-factor *wR* and goodness of fit *S* are based on *F*^2^, conventional *R*-factors *R* are based on *F*, with *F* set to zero for negative *F*^2^. The threshold expression of *F*^2^ > σ(*F*^2^) is used only for calculating *R*-factors(gt) *etc*. and is not relevant to the choice of reflections for refinement. *R*-factors based on *F*^2^ are statistically about twice as large as those based on *F*, and *R*- factors based on ALL data will be even larger.

### Fractional atomic coordinates and isotropic or equivalent isotropic displacement parameters (Å^2^)

**Table:**

	*x*	*y*	*z*	*U*_iso_*/*U*_eq_	
C1	0.7961 (5)	0.0222 (7)	0.6839 (2)	0.0317 (8)	
C2	0.6344 (4)	−0.0621 (8)	0.6312 (2)	0.0330 (8)	
C3	0.6332 (5)	−0.2221 (8)	0.5700 (2)	0.0369 (9)	
C4	0.7955 (5)	−0.3400 (8)	0.5399 (2)	0.0369 (9)	
Br1	0.42183 (5)	0.08537 (10)	0.66527 (3)	0.05260 (18)	
O1	0.8584 (3)	−0.1231 (6)	0.73530 (16)	0.0440 (7)	
O2	0.8501 (4)	0.2664 (6)	0.66922 (17)	0.0493 (8)	
O3	0.9456 (3)	−0.2426 (6)	0.56482 (17)	0.0466 (7)	
O4	0.7746 (4)	−0.5294 (6)	0.49066 (17)	0.0482 (8)	
H3A	0.5236	−0.2646	0.5439	0.044*	
H2	0.9357	0.3049	0.7000	0.074*	
H3	1.0256	−0.3258	0.5449	0.070*	

### Atomic displacement parameters (Å^2^)

**Table:**

	*U*^11^	*U*^22^	*U*^33^	*U*^12^	*U*^13^	*U*^23^
C1	0.0278 (18)	0.031 (2)	0.036 (2)	0.0046 (15)	−0.0024 (15)	−0.0024 (17)
C2	0.0275 (17)	0.031 (2)	0.039 (2)	0.0043 (15)	−0.0055 (14)	0.0019 (19)
C3	0.0288 (19)	0.035 (2)	0.045 (2)	−0.0016 (16)	−0.0066 (16)	−0.003 (2)
C4	0.0306 (19)	0.040 (2)	0.039 (2)	−0.0025 (16)	−0.0063 (16)	0.0006 (19)
Br1	0.0335 (2)	0.0616 (3)	0.0615 (3)	0.0137 (2)	−0.00437 (17)	−0.0122 (3)
O1	0.0380 (14)	0.0415 (17)	0.0498 (17)	−0.0045 (12)	−0.0160 (12)	0.0119 (14)
O2	0.0504 (17)	0.0353 (17)	0.0578 (19)	−0.0113 (13)	−0.0256 (14)	0.0076 (15)
O3	0.0332 (14)	0.0487 (18)	0.0578 (19)	−0.0067 (13)	0.0038 (13)	−0.0141 (16)
O4	0.0408 (15)	0.055 (2)	0.0476 (17)	0.0022 (13)	−0.0058 (12)	−0.0205 (15)

### Geometric parameters (Å, °)

**Table:**

C1—O1	1.197 (4)	C4—O4	1.254 (5)
C1—O2	1.299 (5)	C4—O3	1.269 (4)
C1—C2	1.512 (5)	C3—H3A	0.9300
C2—C3	1.302 (5)	O2—H2	0.8200
C2—Br1	1.883 (4)	O3—H3	0.8200
C3—C4	1.475 (5)		
			
O1—C1—O2	125.6 (3)	O4—C4—O3	124.6 (4)
O1—C1—C2	121.3 (3)	O4—C4—C3	117.3 (3)
O2—C1—C2	112.9 (3)	O3—C4—C3	118.1 (4)
C3—C2—C1	126.5 (3)	C2—C3—H3A	118.1
C3—C2—Br1	121.5 (3)	C4—C3—H3A	118.1
C1—C2—Br1	112.0 (3)	C1—O2—H2	109.5
C2—C3—C4	123.9 (3)	C4—O3—H3	109.5

### Hydrogen-bond geometry (Å, °)

**Table:**

*D*—H···*A*	*D*—H	H···*A*	*D*···*A*	*D*—H···*A*
O3—H3···O4^i^	0.82	1.80	2.617 (4)	171
O2—H2···O1^ii^	0.82	1.86	2.681 (4)	176

Symmetry codes: (i) −*x*+2, −*y*−1, −*z*+1; (ii) −*x*+2, *y*+1/2, −*z*+3/2.
